# The volcanic activity changes occurred in the 2021–2022 at Vulcano island (Italy), inferred by the abrupt variations of soil CO_2_ output

**DOI:** 10.1038/s41598-022-25435-4

**Published:** 2022-12-07

**Authors:** Salvatore Inguaggiato, Fabio Vita, Iole Serena Diliberto, Claudio Inguaggiato, Agnes Mazot, Marianna Cangemi, Marco Corrao

**Affiliations:** 1 Istituto Nazionale di Geofisica e Vulcanologia, Sezione di Palermo Via Ugo La Malfa, 153, 90146 Palermo, Italy; 2 Departamento de Geología, Centro de Investigación Científica y de Educación Superior de Ensenada, Baja California (CICESE), Carretera Ensenada-Tijuana 3918, Ensenada, Baja California Mexico; 3 GNS Science Wairakei Research Centre, 114 Karetoto Road, Wairakei, Private Bag 2000, Taupo, New Zealand; 4 Dipartimento di Scienze della Terra e del Mare, Via Archirafi 36, 90123 Palermo, Italy; 5 Geologist, Via Cordovena 50/B, 98071 Capo d’Orlando (Me), Italy

**Keywords:** Geochemistry, Volcanology

## Abstract

The active cone of La Fossa is a close conduit volcano characterized by solphataric activity, manifested by discharging fluids through fumaroles and soil degassing. Since 1978 several degassing crises have been observed and interpreted as early signals of volcanic unrests. Recently, from June 2021 to May 2022, we measured the changes in soils CO_2_ release to evaluating the level and duration of the actual exhaling crises. The CO_2_ output has been evaluated by surveys carried out in anomalously degassing areas, located both in the La Fossa cone summit area and in other peripheral zones, coupled to near-real time monitoring data acquired by three automated stations. The strong and deep input of volatiles released from an underlying magma batch modified the chemical composition of the shallow plumbing system, bringing the system to a higher level of CO_2_ total pressure. This work highlights that a geochemical networks of stations, located at some distance from the fumaroles release and/or from eruptive conduits, is useful and can be applied to characterizing and monitoring any other active volcanic system. This type of studies can be useful to contribute to forecast the next evolution of the studied systems.

## Introduction

One effective approach to investigate the dynamics of volcanoes is monitoring the extensive degassing visible in form of hydrothermal plumes or partially dissolved in ground-water and diffused by the ground^1^. The geochemical monitoring of fluid released is a tool for quantifying the volume of degassing magma, during both active and quiescent phases of volcanic activity^2–15^. The main compounds of extensive degassing are water vapour, carbon dioxide and sulphur species (SO_2_ and H_2_S). Among the volatiles species released by active volcanoes carbon dioxide is generally the most abundant of the dry gases^1^, with worlwide emission of over 50 Tg CO_2_/y^16,17^. Open conduit volcanoes, like Ambryn (Vanuatu) and Mt Etna (Italy) are the top worldwide emitter of CO_2_ realising respectively about 60 and 40 kilotons per day, while emissions from close conduit volcanoes are comprised between 1500 t d^−1^ (Solfatara di Pozzuoli, Italy) and 9 t d^−1^ (Iwoyama, Japan)^1^. Vulcano island, in the Aeolian archipelago (south Mediterranean Sea), is a close conduit volcano that erupted for the last time in 1888–1890. Since then, it has been characterized by a sustained degassing activity, periodically interrupted by degassing crises, as in 1978–1980^18^, 1988–1991^19–23^, 1996^24^, 2004–2007^25–27^, 2009–2010^9–28^, and the last one, still ongoing at the time of writing, started in the second half of 2021^13,29–32^. These crises have been characterized by increasing in volatiles and energy output which suggest a resuming of volcanic activity. In particular, was observed an increase in SO_2_, CO_2_ and H_2_O fluxes from the solphataric plume emission^1,9,13^ corroborate by a general increase in the gas/water ratio of the high temperature fumaroles emissions.

The former recorded anomalous degassing occurred in the 2009–2010 was characterized by an increase of the SO_2_ plume fluxes from 14 to 100 t d^−1^ and by a general increase of diffuse degassing on the upper rim of the active cone. Moreover, the soil CO_2_ fluxes recorded at VSCS station located in the N-E rim of crater area increased from 1000 to 10,000 gm^−2^ d^−1^^9^.

The ongoing crisis, started in late June 2021, showed an abrupt and intense degassing of CO_2_ and SO_2_ occurred in the summit area of the Vulcano edifice^13^ characterized by an increase in deep magmatic fluids component, like SO_2_, CO_2_ and He^33,34^. Moreover, in the half of September, a significant increase in daily frequency of local seismicity and ground deformations, located below the La Fossa Crater, was observed^35^.

**Figure 1:**
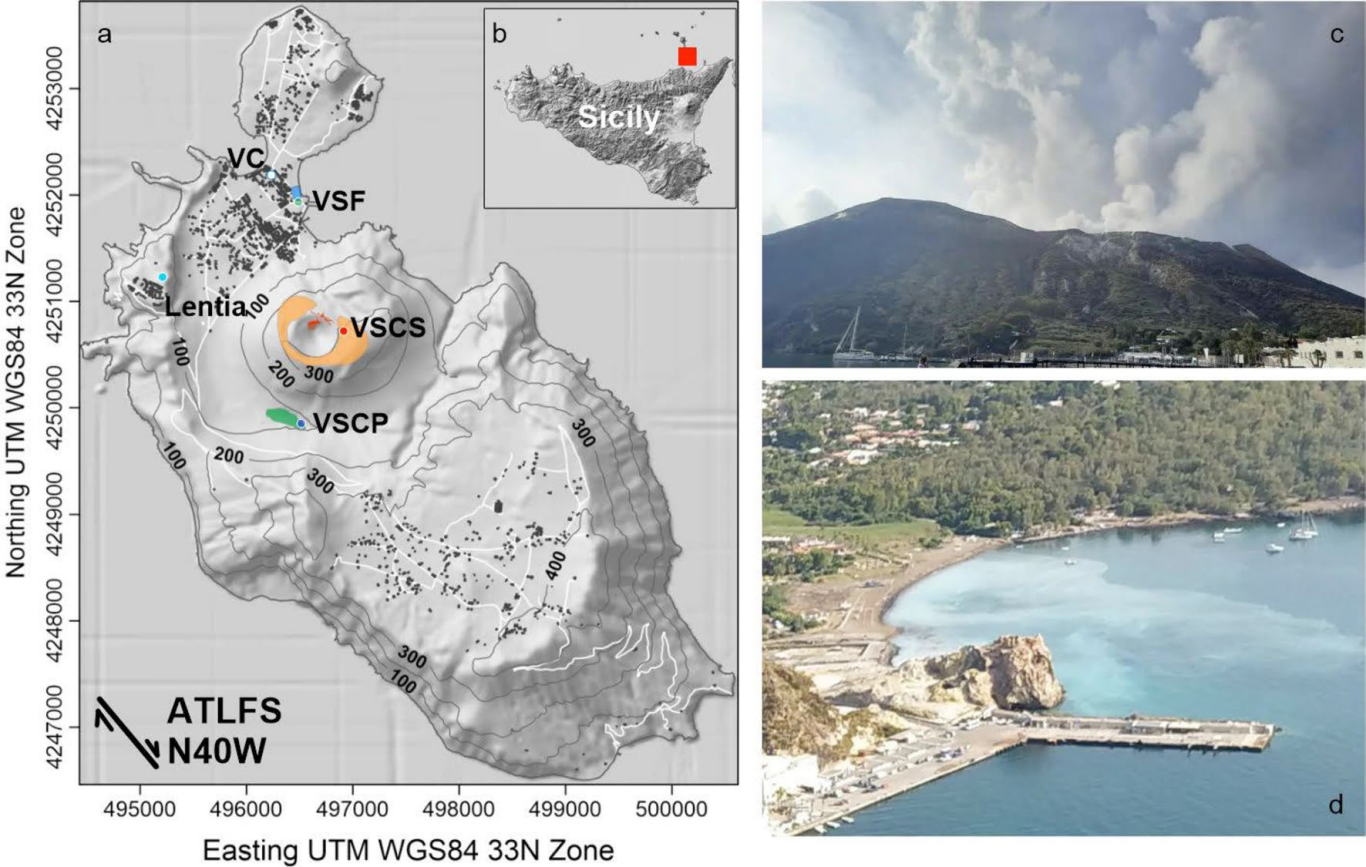
(**a**) Map of the island of Vulcano with the locations of soil CO_2_ fluxes stations (VSCS, VSCP and VSF red, blue and green circles); The anomalous monitored survey areas have been highlighted: La Fossa crater (Brown), Palizzi (Green) and Levante Bay (Blue); Metereological station for local reference of weather condition (Lentia blue light circle); Volcanological Center (VC white circle); Orientation of the Aeolian Tindari-Letojanni Fault systems (ATLFS N40W). (**b**) Map of Sicily Island with the location of the Vulcano Island (red square). (**c**) Picture of the Summit fumaroles showing the strong degassing of the active cone (view from north, September 2021). (**d**) Picture of the Levante Bay area showing the turbidity of sea water due to the strong bubbling degassing (view from south May 2022). The map was generated by Golden Software Surfer (version 24.1.181) and Grapher (version 17.1.408); (http://www.goldensoftware.com).

The fumarolic emissions in the island of Vulcano are present in the summit area of the crater, where a large fumarolic field is active at La Fossa cone with temperatures up to 380 °C, and in the Baia di Levante which is characterized by fumarolic emissions at the boiling temperature in the shoreline and underwater (Fig. 1).

Carbon dioxide is emitted not only by the huge fumarolic field of La Fossa cone, but also by the coastal hydrothermal aquifer in, and through undersea gas bubbling. Moreover, the same convective CO_2_ release degrades in extensive diffuse soil CO_2_ emissions, mainly located on the Levante Bay and Palizzi areas characterized respectively by hot (boiling) and cold (environmental) emission temperatures. The former estimation of the total CO_2_ output from Vulcano was about 500 t d^−1^, as evaluated at the end of the 2004–2007 degassing crisis^1^ and on the light of the overall behavior of this volcanic system we refer to that former estimation as a background.

The main contribution to the total subaerial CO_2_ emissions on the Island is supplied by the fumaroles of La Fossa cone (453 t d^−1^ out of the total background discharge of 500 t d^−1^). Other 20 t d^−1^ were measured from the diffuse soil emissions, 6 t d^−1^ were evaluated from the outgassing hydrothermal aquifer and 4 t d^−1^ was the contribution estimated from the undersea gas bubbling visible in the intertidal and tidal zones^1^.

The aim of this paper is the updated evaluation of the volcanic activity changes based on:

- the near real-time monitoring of soil CO_2_ fluxes diffused at the La Fossa Cone and the peripheral areas of Palizzi and Levante Bay;
- discontinuous monitoring of CO_2_ fluxes diffused by soil in areas around the CO_2_ continuous monitoring stations, La Fossa Cone, Palizzi and Levante Bay.

Moreover, the near real time and discontinuous monitoring of CO_2_ fluxes diffused by soil in summit and peripheral volcanic areas allowed us to model the changes of volcanic gas propagation (mainly CO_2_) from the deep plumbing system to the summit and peripheral volcanic areas, before, during and after the unrest phase.

## Methods

### Soil CO_2_ flux network

Near continuous soil CO_2_ fluxes measurements have been carried out by a network system covering three main anomalous degassing areas of the Vulcano island^1,9^. In particular, three station are located respectively in the Summit, Palizzi and Levante Bay areas: VSCS, VSCP and VSF (Fig. 1; Tab. 1 in supplementary information). These stations are aligned on the NW direction passing through the central crater area. They are manufactured by West Systems S.r.l., and perform the dynamic accumulation chamber method^33^. The accumulation chamber method is based on the principle of the measurements of the increasing CO_2_ concentration in the time [CO_2_]/dt inside of the closed chamber of known volume placed face-down on the soil. The CO_2_ accumulation rate is used to calculate the flux of gas through the soils expressed in g m^2^ d^−1^. Carbon dioxide is measured with a Dräger Polytron IR spectrometer, which operates in the range of 0–9999 ppm (precision of ± 5 ppm). The environmental parameters (wind direction and speed, soil and atmosphere temperatures, atmospheric pressure and soil and atmosphere relative humidity) are acquired at the same time and in the same place^9^ to fit each flux evaluation to the different atmospheric conditions. The near-real-time measurements of CO_2_ fluxes network are carried out on an hourly basis and data are stored in-situ and transmitted directly to the INGV–Palermo geochemical monitoring center, via internet utilizing the WLAN/rooter service^9^. In case of failure in the transmission system, the local storage prevents the loss of data and the time series can be updated by in situ downloading.

The high wind speed influences the soil CO_2_ flux measurement, producing turbulence in the shallow part of the soil, disturbing the concentration/pressure gradient responsible of the flow of gases towards the atmosphere, making impossible in this specific case to estimate the flow of CO_2_. The CO_2_ fluxes presented in this study were revised and selected on the basis of the wind speed and the R^2^ of the accumulation rate measurements curve. All the CO_2_ fluxes acquired when the wind speed was lower than 6 m/s and the R^2^ accumulation rate curve higher 0.99 were considered in this study.

Moreover, the probability statistical Pearson test was applied at our dataset to investigate the significative correlation between the environmental parameters (temperature and pressure) and the CO_2_ fluxes acquired by the stations of the continuous network (VSCS, VSCP and VSF). The results showed a not significative correlation (p-value > 0.05) for VSCS, VSCP and VSF between these parameters with p-values respectively of 0.154, 0.864, 0.648 for temperature-CO_2_ flux; and respectively of 0.154, 0.267, 0.799 for pressure-CO_2_ flux.

### Soil CO_2_ output surveys

Soil diffuse CO_2_ flux surveys have been carried out in three different areas of Vulcano: the summit area of La Fossa crater, Palizzi and Levante Beach areas. Soil CO_2_ flux was measured using the accumulation chamber method and the increase of CO_2_ concentration was analyzed by a portable non-dispersive infrared systems (WS-LI820-CO2: West Systems S.r.l., Pontedera, Pisa, Italy). All measurements were performed under dry conditions and stable atmospheric pressure, in order to reproduce similar exposure conditions of the un-saturated shallow ground, minimizing the eventual effects due to occasional variables of external origin (e.g. rainfall, windstorm). The measurement method is described in detail in^36^. In the experimental text made by^37^ the mean difference between the CO_2_ flux measurement by the accumulation chamber method and the imposed flux was estimated to be − 12.5%.

Repeated CO_2_ flux surveys in the above mentioned areas have been performed from 2007 to May 2022. Depending on the surveyed area, different numbers of CO_2_ flux measurements were carried out, and they are listed in Table 2 (supplementary information). The graphical statistical analysis (GSA) was performed to process the CO_2_ flux. GSA permits differentiation of the populations of the CO_2_ flux data, when they correspond to different Gaussian dispersion around their characteristic mean of flux values^36,38,39^. We partitioned the CO_2_ flux data into different lognormal populations and estimated the proportion, mean, and standard deviation of each population, following the procedure outlined by^40^. An interpolation algorithm for sequential Gaussian simulation was applied to mapping the three degassing areas and estimating the total CO_2_ emission, with the associated uncertainty^41^. The basic idea of the sequential Gaussian simulation (sGs) is to generate a set of equiprobable representations (100 realizations for this study) of the spatial distribution of the simulated values, reproducing the statistical (histogram) and spatial (variogram) characteristics of the original data. The CO_2_ fluxes, measured from the randomly distributed measurement points, were interpolated by a distribution over a grid of square cells covering the study areas using the exponential variogram model^41^. The produced variograms for any output survey are reported in Fig. S1 (supplementary information). The measured CO_2_ fluxes in randomly distributed points on the surface were interpolated by a distribution over a grid of square cells whose length resulting from the average distance between measurement points: 5 × 5 m^2^ in the Summit area; 2 × 2 m^2^ in the Levante Bay area; 10 × 10 m^2^ in the Palizzi area). Then, 100 simulations of the CO_2_ fluxes with the obtained distribution were performed. For each simulation, the CO_2_ flux estimated at each cell is multiplied by the surface covered by one square cell (25 m^2^ for la Fossa crater, 4 m^2^ for Levante Bay area and 100 m^2^ for Palizzi area) and added to the CO_2_ fluxes estimated at the neighborhood cells to obtain the total CO_2_ output. The CO_2_ output diffuse from soil in the summit area has been extended to cover the La Fossa crater rim (70,575 m^2^), except the sector covered by the fumarole vents. The surveys on the Levante Bay and Palizzi areas covered 7200 m^2^ and 19,300 m^2^, respectively. The differences among all simulated maps are used to compute the uncertainty of the flux estimation, according to^42^.

## Results

### Discontinuous variations of soil CO_2_ output of Crater area

**Figure 2:**
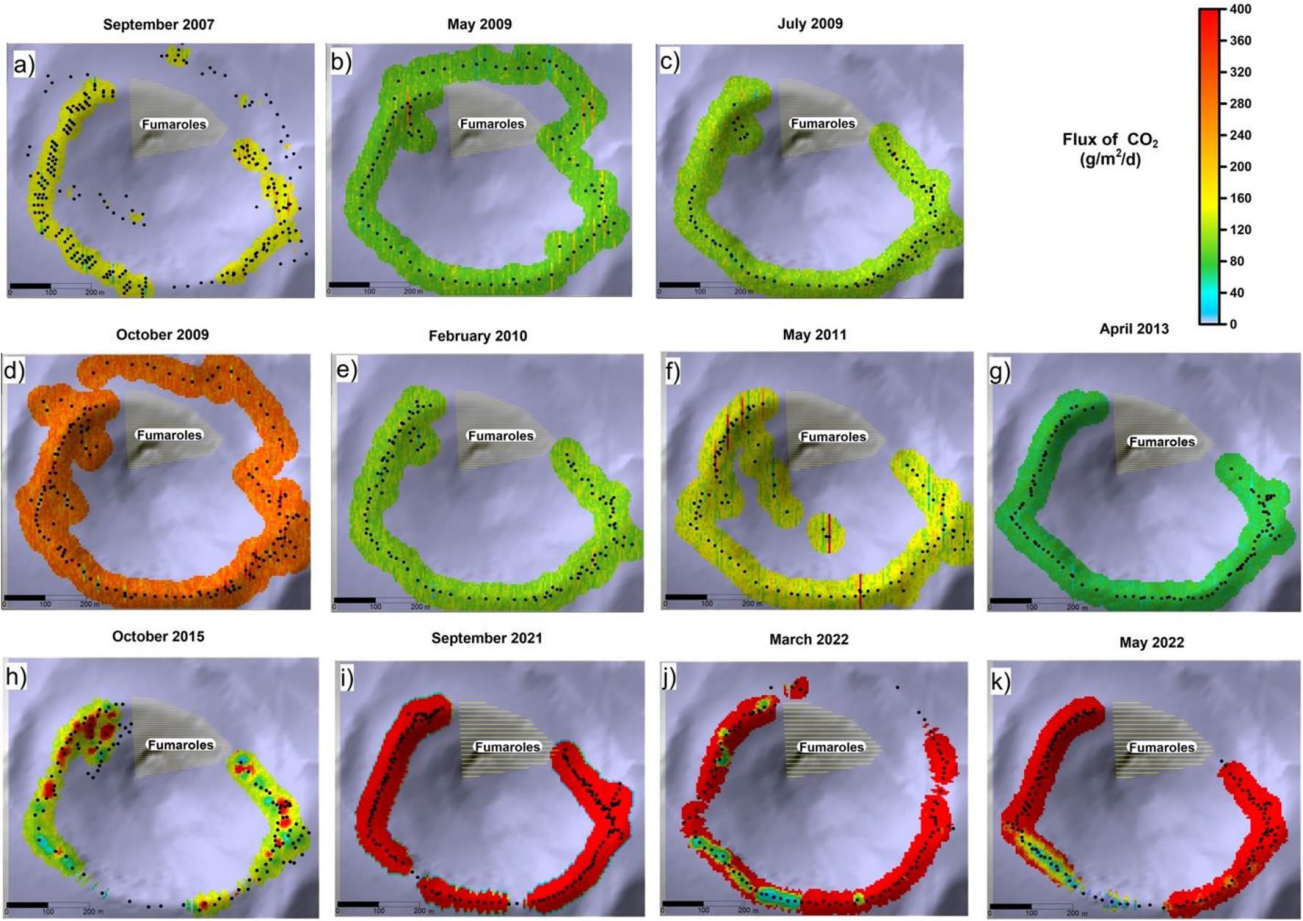
Soil CO_2_ flux maps of summit area (in g m^−2^ d^−1^) obtained by the sGs 100 sequential Gaussian simulations from September 2007 to May 2022. The blanked area interested by intense fumarole emissions is also indicated. The maps were generated by Surfer Software Version 11.6.1159 Surface Mapping System (http://www.goldensoftware.com).

The results of the discontinuous CO_2_ flux surveys of Crater area, performed from 2007 to 2022, are summarized in Table 2 (supplementary information) and Fig. 2. Eleven surveys have been carried out in the summit ring of the active cone and the maps resulting from the sequential Gaussian simulations can be compared in Fig. 2a–k.

The extension of each survey around the soil CO_2_ monitoring station “VSCS” (and the spacing between measurement points) varied from survey to survey (see Table 1 in supplementary information). It was depending on the exposure of the anomalous degassing ground surface, and for safety reasons related to the hostile condition caused by the intense release of hot and noxious gases from the neighboring fumaroles. In each survey, a surface ranging from about 70,000 to 198,000 square meters was covered. The output of soil CO_2_ flux in crater area was calculated by the sequential Gaussian simulation for each survey and standardized to the smallest area calculated of (70,755 m^2^) using the follow equation:

1 $${\text{Total CO}}_{{2}} \,{\text{output }} = {\text{ TCO}}_{{2}} \_{\text{SGS}}\_{\text{covered area }}* \, \left( {{\text{Area}}_{{{\text{Smallest}}}} /{\text{Area}}_{{{\text{Covered}}}} } \right)$$

The total CO_2_ output from 2007 to 2021 ranged from 8 t d^−1^ (April 2013) to 248 t d^−1^ (September 2021). From 2007, 4 periods of increased gas emissions occurred, in 2009, 2011, 2015 and second half of 2021. In the second half of 2021 the CO_2_ flux output was the highest, reaching in September 2021 the maximum value ever measured (248 t d^−1^).

### Discontinuous variations of soil CO_2_ output of Palizzi area

**Figure 3:**
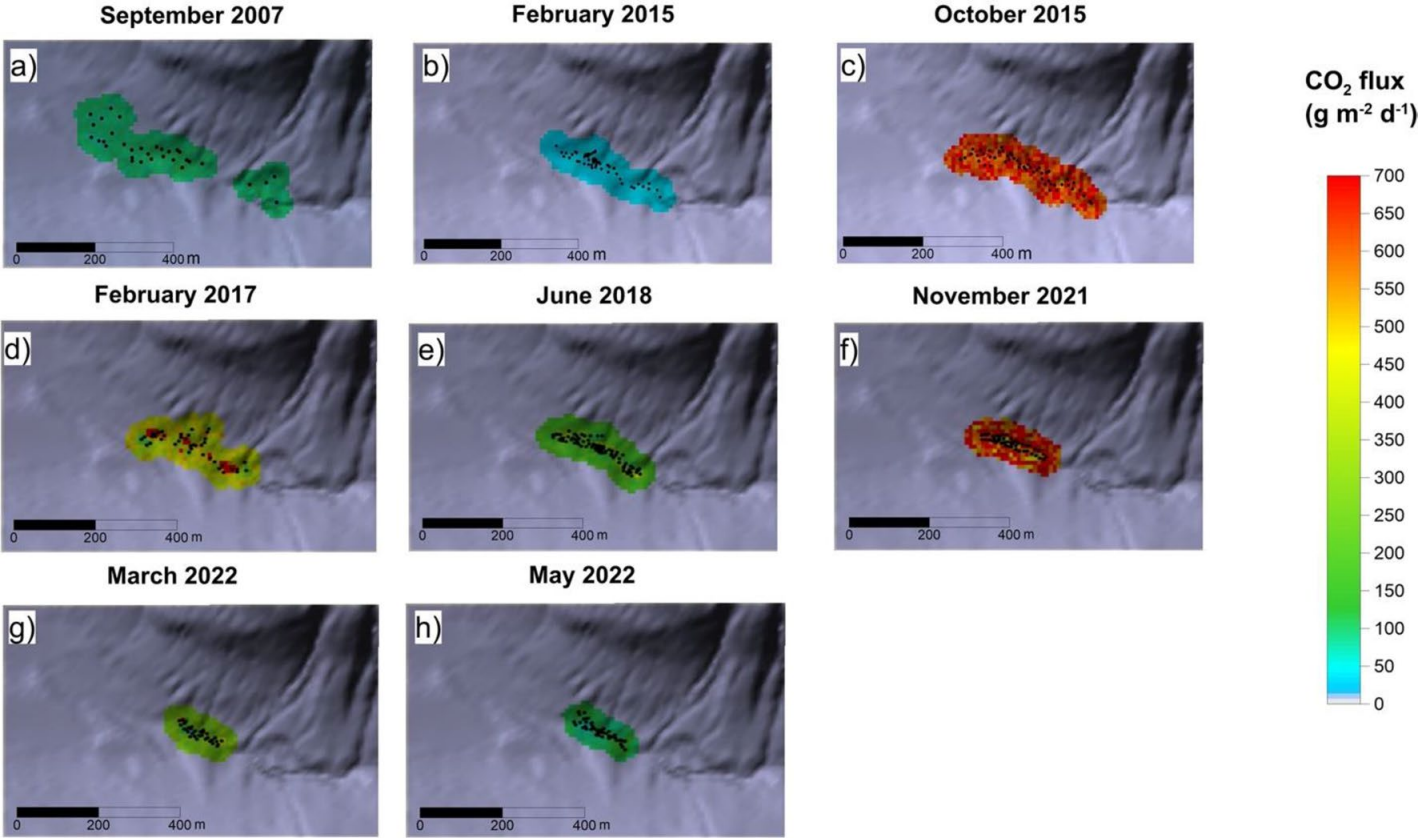
Soil CO_2_ flux maps of Palizzi area (in g m^−2^ d^−1^) obtained by the sGs from September 2007 to May 2022. The black dots are measurement points. The maps were generated by Surfer Software Version 11.6.1159 Surface Mapping System (http://www.goldensoftware.com).

From September 2007 to May 2022, eight CO_2_ flux surveys around the soil CO_2_ monitoring station “VSCP” have been performed. The results are listed in Table 1 (supplementary information) and Fig. 3a–h.

Soil CO_2_ output was calculated by the sequential Gaussian simulation for each survey and standardized to the smallest area of 19,300 m^2^, using Eq. (1).

The total CO_2_ output from 2007 to 2021 ranged from 0.8 (February 2015) to 31 t d^−1^ (November 2021). High CO_2_ outputs occurred in October 2015 (16.9 t d^−1^), February 2017 (18.5 t d^−1^) and November 2021 (31 t d^−1^). The highest CO_2_ output occurred both at Palizzi and Crater areas in the second half of 2021.

### Discontinuous variations of soil CO_2_ output of Levante Bay area

**Figure 4:**
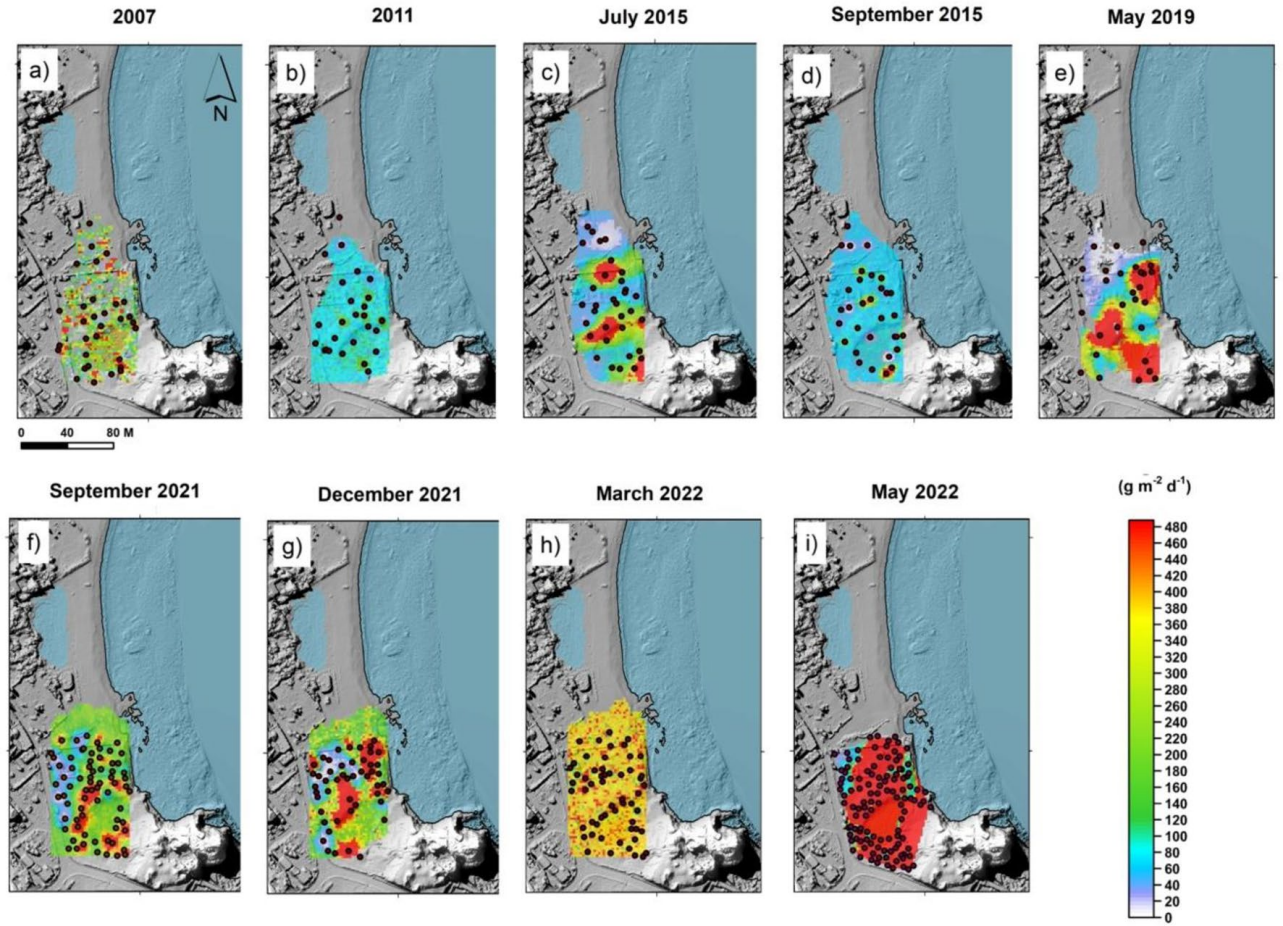
CO_2_ flux maps of Levante Bay area (in g m^−2^ d^−1^) obtained by the sGs from September 2007 to May 2022. The black dots are measurement points. The maps were generated by Surfer Software Version 11.6.1159 Surface Mapping System (http://www.goldensoftware.com).

Nine soil CO_2_ flux surveys have been performed from September 2017 to May 2022 in Levante Bay area. The investigated area includes the soil CO_2_ monitoring station “VSF”, located close to the mud pool exploited for touristic thermal bathing. The results are summarized in Table 1 (supplementary information) and Fig. 4a–h.

Soil CO_2_ output for each survey was calculated by the sGs and standardized to the smallest area of 7200 m^2^, using Eq. (1). The total soil CO_2_ output from 2007 to 2021 ranged from 1.2 t d^−1^ (May 2011) to 16.4 t d^−1^ (May 2022). The CO_2_ flux values have increased from 2015 to 2022. The CO_2_ output increased one order of magnitude respect to the base level (2017) reaching the maximum values of 14.3 and 16.4 t d^−1^, respectively in December 2021 and May 2022. The total CO_2_ output had slightly decreased in March 2022, but afterwards high values of CO_2_ flux have been measured again in the studied area (Fig. 4).

### Near real time variations of soil CO_2_ fluxes

**Figure 5:**
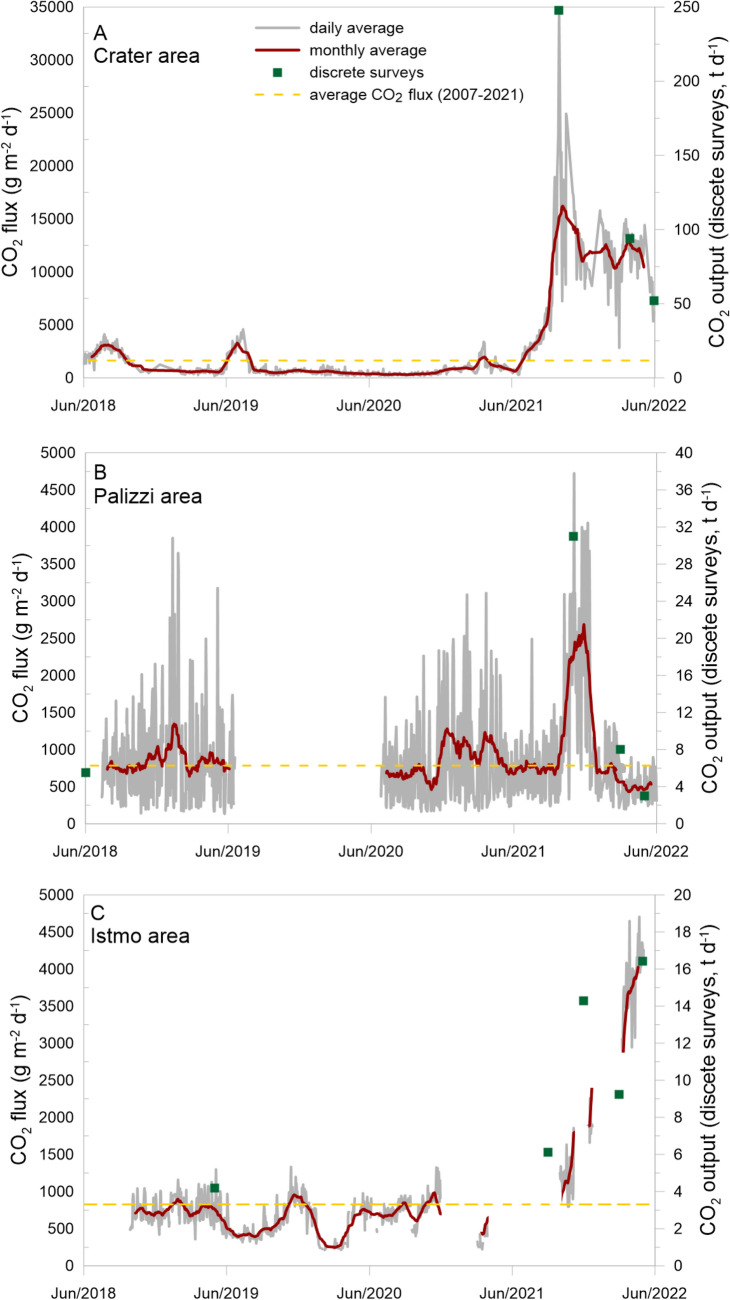
Time variation of soil CO_2_ fluxes from Summit, Palizzi and Levante Bay stations (VSCS, VSCP and VSF). The yellow dotted line indicates the local background degassing levels; the red curve shows the monthly running average of recorded flux values at the different location; Each green square symbol stands for the CO_2_ output evaluated by the surveys carried out around each monitoring stations. The monthly average recorded at each station highlights the significant delays registered within the monitoring network on a monthly time scale, by filtering out the minor short-term effects (**A**–**C**). The highest data values of CO_2_ fluxes were recorded respectively in September 2021, December 2021 and May 2022. Clearly state the continuous surveys do not allow total flux calculations of the area but only provide information of the flux in one location (g m^−2^ d^−1^) while the discreet surveys provide total fluxes of the region over which they were made. The comparison between these two parameters however detected if the variations observed in the continuous monitoring on a single point are congruent with the areal variations, and confirm that when consistent increases in degassing occur they affect the whole surrounding area where the monitoring station is located. Obviously, the different frequency of acquisition of these two parameters determines that the discrete survey can lose information and significant variations of degassing activity. Indeed, speculation about the delays in the evolution of the system between the different observation sites has been based essentially on near-continuous data.

The fluxes of CO_2_ from the ground hourly recorded by the VSCS station tracked the changes of the CO_2_ degassing rate in the summit area of the island of Vulcano in the period 2018–2022 (Fig. 5). The summit monitoring station (VSCS) showed, values ranging between 300 and 34,000 g m^−2^ d^−1^. The previous monitoring period was used to assess the background range typical in the active cone area^13^, we considered the soil CO_2_ flux value of 1720 g m^−2^ d^−1^ as the background degassing level calculated as the average of hourly recorded values before the last crisis (from 2018 to June 2021) (Fig. 5A).

Starting from June 2021 onwards, the CO_2_ diffuse gas emissions at VSCS station showed a positive trend, with an abrupt increase, lasted until September 2021. In September, the soil CO_2_ fluxes actually increased about 20 times respect to the previous background, remaining above 10,000 g m^−2^ d^−1^ in the summit area (VSCS), until May 2022.

The VSCP monitoring station, located at the southwestern foot of the cone, showed soil CO_2_ flux values ranging from 220 to 4700 g m^−2^ d^−1^, with a background reference level of 980 g m^−2^ d^−1^ calculated averaging the values locally recorded from 2018 to September 2021 (Fig. 5b). A large increase in the CO_2_ fluxes was evident only in October 2021, with the highest values of 4724 g m^−2^ d^−1^ recorded on first November 2021. Thereafter the degassing values decreased rapidly, getting back to values of 820 g m^−2^ d^−1^, lower than the local background reference level.

The VSF monitoring station located in the Levante Bay showed values between 212 and 5146 g m^−2^ d^−1^, with a local background degassing level of 647 g m^−2^ d^−1^ calculated as the average of hourly recorded values before the last crisis (from 2018 to June 2021) (Fig. 5c). In this location a large increase in the diffuse CO_2_ fluxes from the ground was recorded in October 2021, but significant anomalous degassing values, over 4000 g m^−2^ d^−1^ were reached after April 2022, with the highest value recorded on May 2022 (5146 g m^−2^ d^−1^).

## Discussion and conclusions

Soil CO_2_ fluxes emitted at Vulcano Island, have been monitored both in a continuous mode and in discontinuous in the three target areas, highlighting the significant variations occurred in the solphataric activity, during the last period of observation 2021–2022. The periodical surveys carried out around the monitoring stations verified the areal extension of the outgassing anomaly and allowed to determine the partial contribution of degassing CO_2_ from each of the three target area.

**Figure 6:**
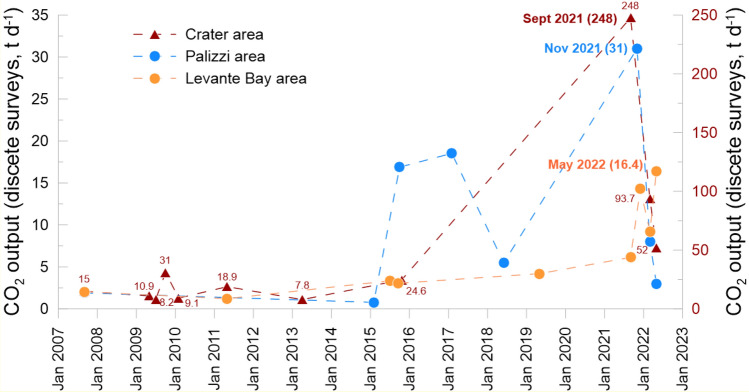
Time variation of soil CO_2_ output from Crater and peripheral areas of Levante Bay and Palizzi evaluated by periodical surveys (red triangles, brown circle and blue circles respectively). The maximum outgassing occurred in September 2021 at Crater area with 248 t d^−1^ and in November 2021 at Palizzi area with 31 t d^−1^, while the outgassing peaked in May 2022 at the Levante Bay area with 16.4 t d^−1^.

In particular, the strong and abrupt increase of diffuse degassing started both in the summit and peripheral areas, during the second half of 2021, showing soil CO_2_ output values never measured before in the same areas. In the period September 2021–May 2022, the CO_2_ flux increased 32, 31 and 15 times compared to the local background values, respectively in the Crater, Palizzi and Levante Bay areas (Figs. 2, 3, 4) and Table 1 (supplementary information). The time variations of CO_2_ output evaluated in the three target areas are plotted on Fig. 6. The Fig. 6 shows the sharp and strong degassing rates, started in the second half of 2021 and in the first half of 2022, respectively at Crater, Palizzi and the Levante Bay areas. The Crater area reached the highest CO_2_ output in September 2021 (248 t d^−1^) while the highest output from the Levante Bay area occurred in May 2022 (16.4 t d^−1^) and both areas are still showing degassing levels higher than the background references previously identified. Differently, the Palizzi area reached its maximum output in November 2021 (31 t d^−1^), getting back to its background values already in March 2022.

**Figure 7:**
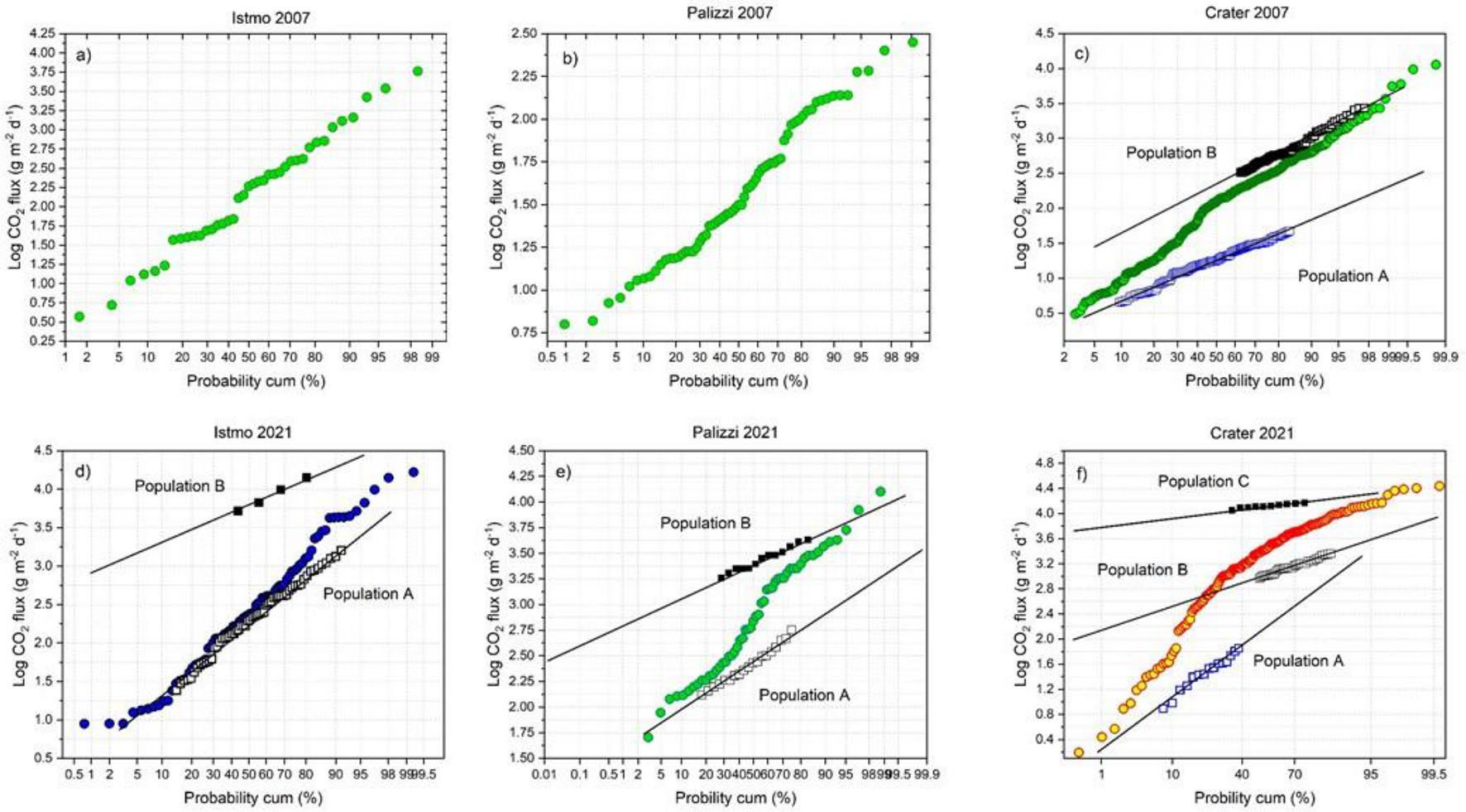
Log CO_2_ fluxes vs Cumulated probability (%) for the 2007 and 2021 surveys at anomalous target areas: (**a**,**d**) South sector of the Levante Bay; (**b**,**e**) Palizzi; (**c**,**f**) Summit ring of the active crater.

The probability plots resulting from the flux measured in the three areas (Levante Bay, Palizzi and La Fossa Crater) respectively in September 2007 and September 2021 (lower vs higher degassing stage) show different log-normal distributions (Fig. 7). They show an increase of both number of degassing family and their mean fluxes (Table 3 supplementary information), allowing to identify the changes in level and degassing style in all the investigated areas. In particular, the Palizzi and Levante Bay areas showed a log-normal distribution, with only one population in 2007 and two populations in 2021 with a strong increase of degassing values (Fig. 7a,b and d,e). At La Fossa Crater area, the log-normal distributions showed two populations in 2007 and three populations in 2021 (Fig. 7c and f), with a very strong output variation, and the mean value of the highest degassing family increasing about thirty times, that is from 455 to 13,518 g m^2^ d^−1^. In this study, the different populations correspond to the soil respiration caused by the biological background, the hydrothermal CO_2_ degassed from groundwater, and the soil gas emissions surrounding fumaroles conduits. The isotopic composition of δ^13^C_CO2_ of soil gases in the peripheral areas of Vulcano corroborate the strong increase of deep-origin CO_2_ began in the September 2021 and reaching the maximum to November 2021^43^.

Utilizing the above mentioned GSA methodology, a Total Output of CO_2_ for the three investigated areas have been estimated and compared with sGs methods for La Fossa Crater, Palizzi and the Levante Bay areas. The results (Tab. 4, supplementary information) show good correspondences between these two methodologies.

The near continuous monitoring of soil CO_2_ fluxes, carried out in the same areas, confirmed relevant variations in soil degassing, increasing by 20, 6 and 8 times higher than the background level respectively, in the VSCS, VSCP and VSF stations between September 2021 and May 2022 (Fig. 5).

The sequence of variations and timing of the CO_2_ output from the different areas have been compared to the near continuous monitoring of soil CO_2_ fluxes allowing to investigate in detail the delayed responses of the shallow system to the deep volatiles input (see Fig. 6). The anomalous shallow release of soil CO_2_ begins at the crater station (VSCS) in June 2021 and reaches the maximum values of 34,000 g m^2^ d^−1^ in September 2021 (Fig. 5A). In the peripheral areas, the VSCP and VSF stations registered the increase of CO_2_ flux from October 2021, that is about 4 months later than the summit area (Fig. 5B,C). From this observation, we propose that the deep CO_2_ input, that firstly reached the summit area, showed a delay in the peripheral areas, due to the different propagation time between the main fumaroles conduits and the peripheral zones of the shallow plumbing system, variously interacting with the underlying and multilayered aquifers (Fig. 5A–C). Moreover, these peripheral areas showed a different degassing rate, with the maximum value of CO_2_ flux reached in November 2021 at Palizzi and in December 2021 at the VSF station (Figs. 5, 6). After that, the VSCP station went back to the background value in March 2022, while the VSF station has maintained an anomalous degassing level, up to the first months of 2022 reaching the highest values of CO_2_ degassing at May (Figs. 5B,C, 6). The different geo-hydrological features of Palizzi and Levante Bay account for the different degassing behavior observed in these two areas. The Palizzi area lies on an underlying cold aquifer receiving the cold meteoric recharge mainly from the Piano area^1,44^. The Levante Bay area lies on an underlying powerful thermal and saline aquifer, which is recharged by waters of both meteoric and sea origin, and which has strong buffering power^45,46^.

The anomalous diffuse degassing at the Levante Bay area (VSF), showed a significant delay, respect to the crater area, being manifest about 4 months after to the starting input that affected the VSCS station. Moreover, the Levante Bay is spending more time (5 months more than the Palizzi area) to discharge the increased CO_2_ input and to reach the highest degassing value and has not yet recovered the former background level. The delay, observed in the Levante Bay area, could be ascribed to multiple sequences of buffering and scrubbing processes, caused in this area by the presence of a huge and multi-layered hydrothermal system^45^. The scrubbing process could be explained considering a former slow and continuous process of CO_2_ dissolution in the shallow hydrothermal system, occurred in June-December 2021^13,47^ which brought to a complete saturation. Therefore, any further CO_2_ dissolution is now greatly lowered or even impeded, and any new deep input can flow through the aquifer without significant interactions, allowing the new deep CO_2_ input to rise directly to the surface^48^. Consequently, more gas emission through the soil has been registered at the Levante Bay area (VSF), and the bubbling gas rates has visibly increased at the same time in the intertidal zone (Fig. 1d), and in the mud pool.

**Figure 8:**
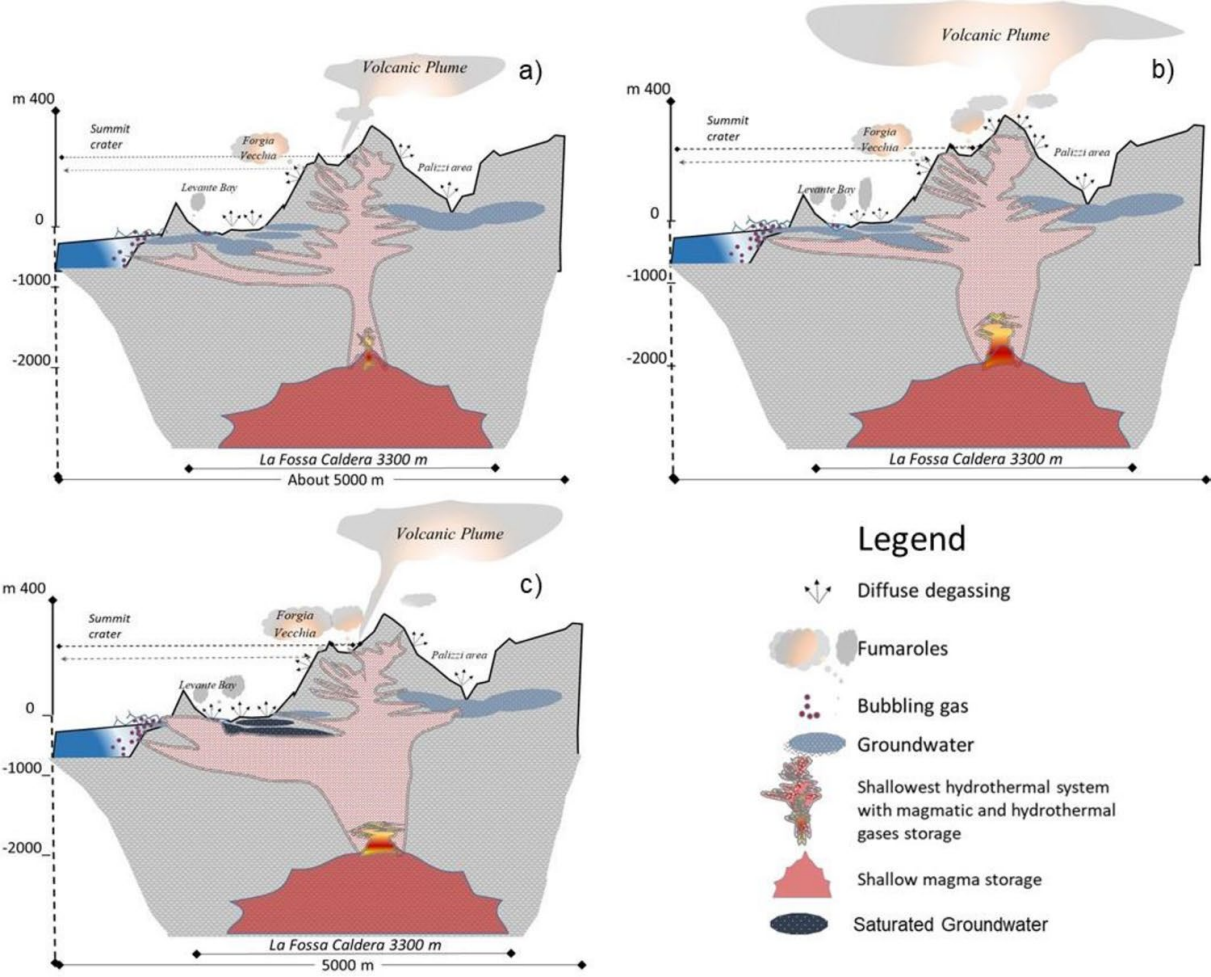
Sketch map of Vulcano plumbing system of NW–NE profile of the Caldera La Fossa running from Mt. Lentia, Vulcano Piano. The ratio between height and length of this profile is H/L = 1.5; the total length is 4.6 km; and La Fossa Caldera occupies 3.3 km along this section. The schematic sections show: (**a**) the background condition of solphataric release (modified from Inguaggiato et al.^13^); (**b**) a starting anomalous deep input that influenced the fluid release at summit area of the active cone and at the Palizzi area. (June–December 2021); (**c**) the volatiles wave propagation arrived at the Levante Bay area (January–May 2022), when the deep input of mass and energy modified the buffer and scrubbing property of the underling hydrothermal system triggering the maximum CO_2_ output ever recorded. The maps were created using Microsoft^®^ PowerPOint^®^ 2016 (16.0.4266.1001) MSO (16.0.5365.1000). (https://www.microsoft.com/it-it/microsoft-365/previous-versions/microsoft-office-2016).

In conclusion, the deep input of volatiles, released from the new and rich magma batch^13,33–35,43^, strongly modified the physico-chemical composition of the shallow plumbing system below the Levante Bay, affecting the total pressure of CO_2_ which brings the system to a higher potential energy than showed in previous decades. The continuous monitoring data, coupled to repeated systematic surveys, highlighted the delayed effects related to the local chemo-physical condition of intercalated aquifers. The schematic model of the solphataric degassing of the Vulcano island has depicted the different degassing behaviors during the observation period (Fig. 8). The preferential degassing areas (Fig. 8) have been affected by a different and intensive increase in the energy and mass fluxes transferred to the surface^13^. Actually, the coastal environment of the Levante Bay appears as the most compromised by increased and extensive surface degassing. During this crisis the shallow plumbing system released a great amount of fluids, over an order of magnitude higher than the local background, in order to maintain the dynamic balance between the increasing deep volatiles input and the consequent surface output. The geochemical volcano monitoring deserves, in this period, more attention for identifying any signal of new deep input, which could cause such a delicate dynamic equilibrium to reach and overcome its critical condition, eventually disposing the excess energy and mass in a single solution and producing some paroxysmal event.

## Supplementary Information

Supplementary Information.

## Data Availability

The dataset used during the current study available from the corresponding author on reasonable request.
