# Supplementary material for: The volcanic activity changes occurred in the 2021–2022 at Vulcano island (Italy), inferred by the abrupt variations of soil CO2 output
Source: Sci Rep. 2022 Dec 7;12:21166. doi: 10.1038/s41598-022-25435-4 (PMC9729569; doi:10.1038/s41598-022-25435-4)

# **Supplementary information**

Table 1: CO_2_ soil fluxes network of Vulcano Island, detailed information.

| Name of CO_2_ flux station | Acquisition frequency | Location | Latitude | Longitude | Year of installation |
| --- | --- | --- | --- | --- | --- |
| **VSCS** | Hourly | NE Crater Rim | 38.404081 | 14.965162 | 2007 Sept. |
| **VSCP** | Hourly | Palizzi Area | 38.397505 | 14.958200 | 2017 Jan. |
| **VSF** | Hourly | Baia Levante Area | 38.415739 | 14.959785 | 2018 Apr. |

Table 2. Soil CO_2_ flux measurements number, statistics (average soil CO_2_ flux, standard deviation, minimum and maximum CO_2_ flux), total soil CO_2_ output and standard deviation from sGs and the area covered. The normalized output from La Fossa crater has been extended to cover the crater rim, except the fumarole area and it is 70,575 m^2^. The soil CO_2_ output of Palizzi was standardized to an area of 19,300 m^2^, while the total soil CO_2_ output of Levante beach was standardized to an area of 7,200 m^2^.

|  | **Date** | **Number of  measurements** | **Average  CO_2_ flux (g m^-2^ d^-1^)** | **Standard Deviation  (g m^-2^ d^-1^)** | **Minimum  CO_2_ flux (g m^-2^ d^-1^)** | **Maximum CO_2_ flux (g m^-2^ d^-1^)** | **Total CO_2_ output from sGs (t d^-1^)** | **Standard Deviation  (t d^-1^)** | **Covered Area (m^2^)** | **Total sGs  CO_2_ output  (t d^-1^) Standardized smallest area** |
| --- | --- | --- | --- | --- | --- | --- | --- | --- | --- | --- |
| **La Fossa** | Sep 07 | 244 | **370** | 4.39 | 0 | 11291 | 15 | 0.6 | 70,575 | **15** |
| **Crater** | May 09 | 100 | **161** | 1.61 | 0.4 | 2109 | 27 | 0.7 | 174,225 | **11** |
| **Area:** | Jul 09 | 119 | **132** | 1.72 | 0 | 2068 | 15 | 0.3 | 129,350 | **8** |
| **70,575 m^2^** | Oct 09 | 159 | **527** | 4.37 | 0 | 4207 | 87 | 1.9 | 198,175 | **31** |
|  | Feb 10 | 112 | **154** | 2.33 | 0 | 2633 | 17 | 0.3 | 127,650 | **9** |
|  | May 11 | 101 | **249** | 2.9 | 0 | 1744 | 40 | 1.6 | 151,300 | **19** |
|  | Apr 13 | 113 | **123** | 1.53 | 0 | 1248 | 13 | 0.3 | 113,600 | **8** |
|  | Oct 15 | 125 | **426** | 8.5 | 3.9 | 9854 | 25 | 1.4 | 71,600 | **25** |
|  | Sep 21 | 164 | **4086** | 31.4 | 0 | 27374 | 312 | 7.1 | 88,900 | **248** |
|  | Mar 22 | 111 | **1429** | 17.6 | 0 | 10430 | 111 | 4.3 | 83,400 | **94** |
|  | May 22 | 137 | **879** | 12.0 | 0 | 17696 | 58 | 1.7 | 78,400 | **52** |
| **Palizzi** | Sep 07 | 33 | **169** | 11.3 | 6.3 | 2140 | 5.8 | 0.3 | 57,500 | **1.9** |
| **Area:** | Feb 15 | 58 | **41** | 0.79 | 5.2 | 292 | 1.4 | 0.1 | 34,600 | **0.8** |
| **19,300 m^2^** | Oct 15 | 52 | **868** | 17.5 | 28.7 | 5014 | 38.4 | 2.4 | 43,900 | **16.9** |
|  | Feb 17 | 40 | **1009** | 39.4 | 3.9 | 8142 | 35.9 | 3.1 | 37,400 | **18.5** |
|  | Jun 18 | 83 | **279** | 3.2 | 12.2 | 1496 | 8.9 | 0.5 | 31,300 | **5.5** |
|  | Nov 21 | 53 | **1587** | 42.0 | 4.1 | 12587 | 31 | 9.0 | 19,300 | **31.0** |
|  | Mar 22 | 36 | **407** | 11.5 | 11.1 | 1958 | 7.1 | 0.6 | 17,100 | **8.0** |
|  | May 22 | 50 | **150** | 2.1 | 11.5 | 423 | 2.5 | 0.2 | 16,100 | **3.0** |
| **Levante** | Sep 07 | 31 | **303** | 18.7 | 0 | 2676 | 2.2 | 0.20 | 7,800 | **2.0** |
| **Bay** | May 11 | 28 | **161** | 27.5 | 0.7 | 972 | 1.2 | 0.05 | 7,200 | **1.2** |
| **Area:** | Jul 15 | 35 | **499** | 30.9 | 3.0 | 5782 | 3.8 | 0.40 | 8,100 | **3.3** |
| **7,200 m^2^** | Sep 15 | 32 | **422** | 29.9 | 0 | 4281 | 3.4 | 0.31 | 8,000 | **3.0** |
|  | May 19 | 31 | **774** | 52.7 | 0 | 8023 | 5.2 | 0.54 | 8,900 | **4.2** |
|  | Sep 21 | 70 | **993** | 32.5 | 8.9 | 14083 | 7.75 | 0.63 | 9,100 | **6.1** |
|  | Dec 21 | 49 | **1979** | 86.9 | 0 | 21564 | 16.7 | 1.1 | 8,400 | **14.3** |
|  | Mar 22 | 59 | **1302** | 29.8 | 0.5 | 8890 | 11.5 | 0.41 | 9,000 | **9.2** |
|  | May 22 | 118 | **2784** | 89.0 | 0 | 107560 | 21.2 | 1.8 | 9,300 | **16.4** |

Table 3: Proportions of each population with the mean CO_2_ flux (in g m^-2^ d^-1^) and the corresponding 90% confidence intervals, obtained by the statistical graphical approach of log-normal distribution, for La Fossa Crater, Palizzi and the Levante Bay areas.

| Survey areas |  | Population of CO_2_ flux | Mean flux of CO_2_  (g m^-2^ d^-1^) | 90% confidence interval (g m^-2^ d^-1^) | Proportion (%) |
| --- | --- | --- | --- | --- | --- |
| La Fossa Crater | 2007 | A | 30 | 28-34 | 40 |
|  |  | B | 455 | 414-512 | 60 |
|  | 2021 | A | 606 | 336-1679 | 29 |
|  |  | B | 1343 | 1150-1629 | 57 |
|  |  | C | 13518 | 12816-14789 | 14 |
| Palizzi | 2007 | A | 56 | 50-65 | 100 |
|  | 2021 | A | 400 | 314-568 | 59 |
|  |  | B | 2848 | 2479-3415 | 41 |
| Levante Bay | 2007 | A | 651 | 322-2493 | 100 |
|  | 2021 | A | 580 | 323-1037 | 90 |
|  |  | B | 8949 | 5122-35,729 | 10 |

Table 4: Total Output of CO_2_ estimated with GSA and sGs methods for La Fossa Crater, Palizzi and the Levante Bay areas. The results show good correspondences between these two methodologies.

| Survey areas | Year | Total CO_2_  output from GSA (t d^-1^) | Total CO_2_ output  from sGs (t d^-1^) |
| --- | --- | --- | --- |
| La Fossa Crater | 2007 | 20 | 15 |
|  | 2021 | 200 | 248 |
| Palizzi | 2007 | 1,1 | 1,9 |
|  | 2021 | 27 | 31 |
| Levante Bay | 2007 | 4,7 | 2 |
|  | 2021 | 10,2 | 14,3 |

Figure S1: Omnidirectional experimental variograms (black dots) (γ) of CO_2_ flux normal scores with the respective spherical variogram models (red lines). The resulting experimental variogram models are used for the sGs processing." a) Crater area; b) Levante Bay; c) Palizzi area.

a)


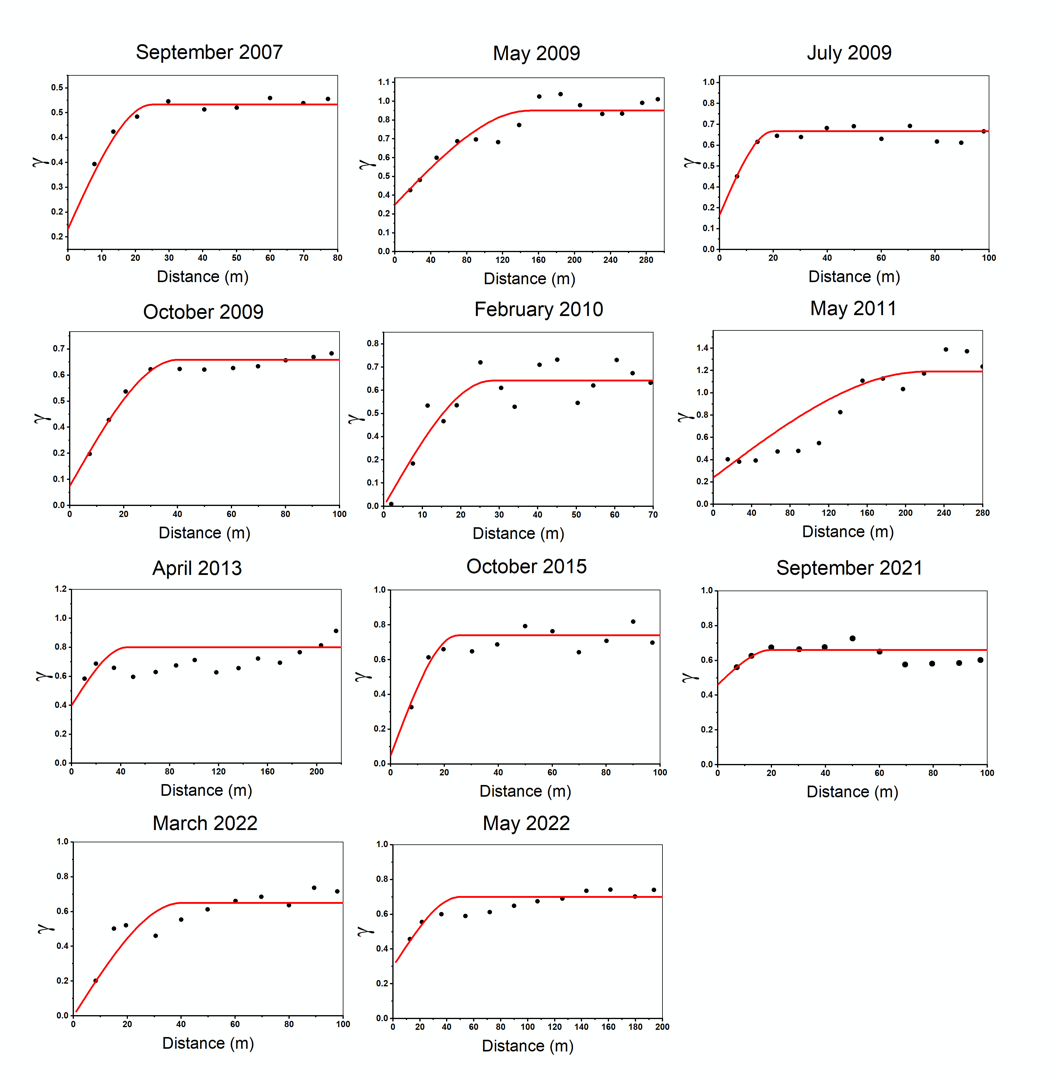


b)


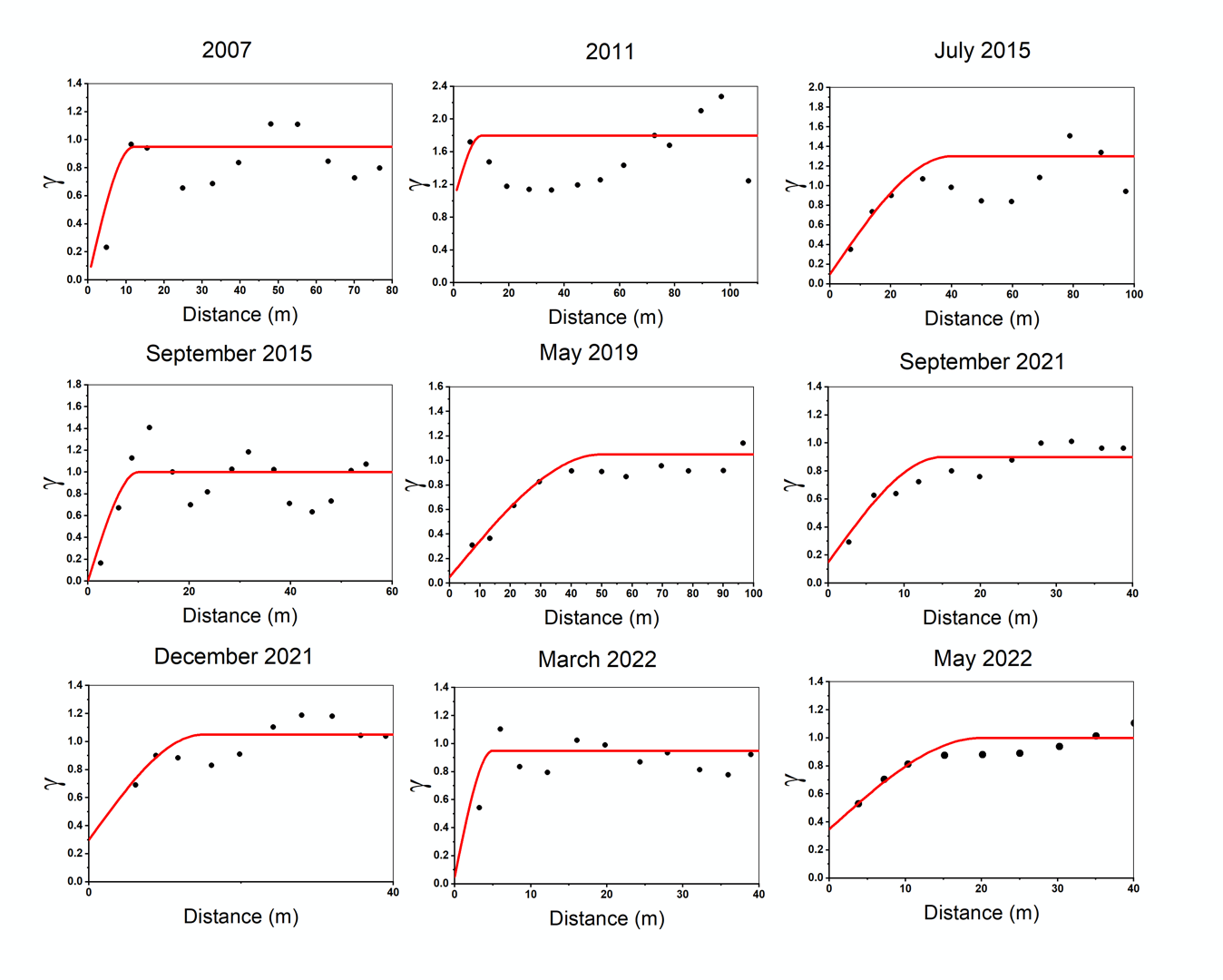


c)


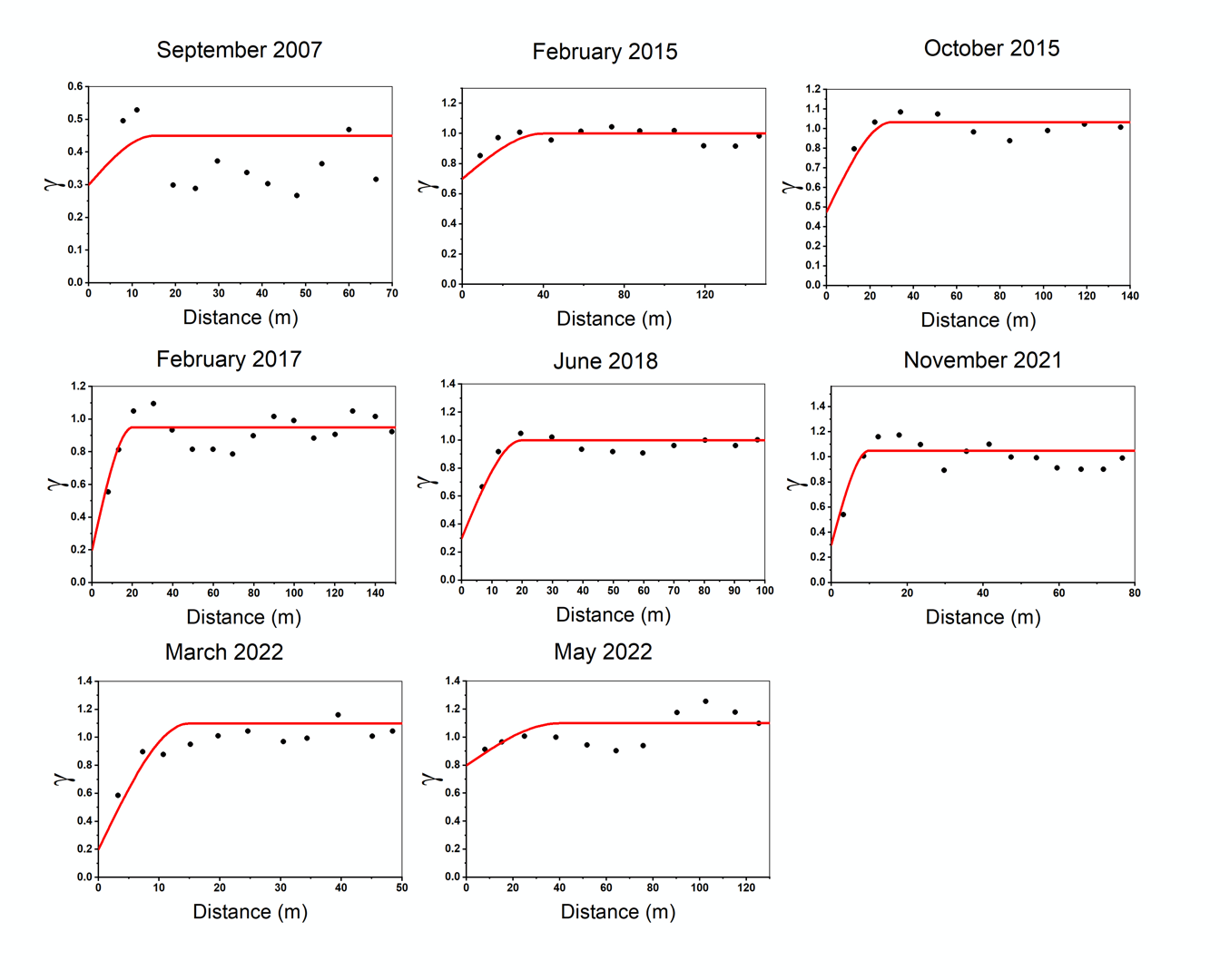

Supplement: Supplementary file 1 — Supplementary Information. [file 41598_2022_25435_MOESM1_ESM.docx]
